# Maternal TET3 is dispensable for embryonic development but is required for neonatal growth

**DOI:** 10.1038/srep15876

**Published:** 2015-10-28

**Authors:** Yu-ichi Tsukada, Tomohiko Akiyama, Keiichi I. Nakayama

**Affiliations:** 1 Division of Molecular Immunology, Medical Institute of Bioregulation, Kyushu University, 3-1-1 Maidashi, Higashi-ku, Fukuoka 812-8582, Japan; 2 Advanced Biological Information Research Division, INAMORI Frontier Research Center, Kyushu University, 744 Motooka, Nishi-ku, Fukuoka 819-0395, Japan; 3 Department of Systems Medicine, Sakaguchi Laboratory, Keio University School of Medicine, 35 Shinanomachi, Shinjuku, Tokyo 160-8562, Japan; 4 Division of Cell Regulation Systems, Medical Institute of Bioregulation, Kyushu University, 3-1-1 Maidashi, Higashi-ku, Fukuoka 812-8582, Japan

## Abstract

The development of multicellular organisms is accompanied by reprogramming of the epigenome in specific cells, with the epigenome of most cell types becoming fixed after differentiation. Genome-wide reprogramming of DNA methylation occurs in primordial germ cells and in fertilized eggs during mammalian embryogenesis. The 5-methylcytosine (5mC) content of DNA thus undergoes a marked decrease in the paternal pronucleus of mammalian zygotes. This loss of DNA methylation has been thought to be mediated by an active demethylation mechanism independent of replication and to be required for development. TET3-mediated sequential oxidation of 5mC has recently been shown to contribute to the genome-wide loss of 5mC in the paternal pronucleus of mouse zygotes. We now show that TET3 localizes not only to the paternal pronucleus but also to the maternal pronucleus and oxidizes both paternal and maternal DNA in mouse zygotes, although these phenomena are less pronounced in the female pronucleus. Genetic ablation of TET3 in oocytes had no significant effect on oocyte development, maturation, or fertilization or on pregnancy, but it resulted in neonatal sublethality. Our results thus indicate that zygotic 5mC oxidation mediated by maternal TET3 is required for neonatal growth but is not essential for development.

DNA methylation, the covalent addition of a methyl group to the C-5 position of cytosine (C), is a common epigenetic modification in most eukaryotes and is implicated in numerous biological processes including genomic imprinting, transcriptional silencing of retrotransposons, and X chromosome inactivation^1^. DNA methylation underlies heritable and long-term silencing of gene transcription that is critical for the development and survival of an organism, and it is therefore considered to be a relatively stable modification compared with other epigenetic marks. However, replication-independent demethylation of DNA has been observed under various circumstances including at specific stages of development^2^,^3^,^4^,^5^,^6^, in association with the transcriptional response to specific signals^7^,^8^,^9^,^10^,^11^, and during artificial reprogramming of differentiated cells^12^. In particular, genome-wide DNA demethylation occurs at two different stages of mammalian development: the migration of primordial germ cells toward the genital ridge^4^,^5^,^6^, and the pronuclear stage of fertilized eggs^2^,^3^. In the latter instance, immunostaining with antibodies to 5-methylcytosine (5mC) revealed a genome-wide loss of 5mC in the paternal pronucleus of mammalian zygotes that begins at sperm decondensation and is marked in extent before replication of DNA, whereas 5mC in the maternal pronucleus was found to be resistant to such depletion^2^,^13^,^14^,^15^. Although the mechanism of replication-independent demethylation of DNA in mammals has been a recurrent subject of uncertainty and controversy^16^,^17^, substantial progress in understanding that of the genome-wide loss of 5mC in the paternal pronucleus of zygotes has been recently achieved. 5-Methylcytosine has thus been found to be oxidized to 5-hydroxymethylcytosine (5hmC), 5-formylcytosine, and 5-carboxylcytosine by the DNA oxidase TET3 in the paternal pronucleus of mouse zygotes, suggesting that the genome-wide loss of 5mC in the paternal pronucleus is attributable mostly to the conversion of 5mC to its derivatives mediated by this enzyme^18^,^19^,^20^. Furthermore, the generated 5mC derivatives are diluted during preimplantation development by DNA replication, similar to the passive DNA demethylation that occurs in the maternal pronucleus^20^,^21^.

The genome-wide loss of 5mC in the paternal pronucleus of mammalian zygotes has been thought to play an important role in mammalian development. This notion is supported by the fact that the phenomenon is conserved among mammalian species including mouse, rabbit, cow, and human^18^,^22^,^23^,^24^. It is also supported indirectly by the observed inefficiency of DNA demethylation in cloned animals^25^,^26^,^27^,^28^. However, other evidence suggests that the genome-wide loss of 5mC in the paternal pronucleus of zygotes is dispensable for mammalian development. Mouse zygotes that were generated by round spermatid injection and in which this process was impaired were thus found to develop normally^29^. In addition, the genome-wide loss of 5mC in the paternal pronucleus is not conserved among all mammalian species, having not been observed in sheep, goat, and pig^30^,^31^,^32^. Thus, in contrast to the advances in understanding the mechanism of this process, its biological relevance remains unclear. Given that the genome-wide loss of paternal 5mC in zygotes has been attributed to the oxidation of 5mC^18^,^19^,^33^, elucidation of the biological relevance of 5mC oxidation may shed light on that of the loss of paternal 5mC. Few studies have examined the role of zygotic 5mC oxidation in mammalian development, however^19^,^34^, although zygotes depleted of maternal TET3 have been generated and studied with regard to elucidation of the molecular mechanism of paternal DNA demethylation^18^,^33^,^34^,^35^,^36^.

We have now evaluated the biological relevance of zygotic 5mC oxidation in mice by depleting oocytes of TET3. We found that attenuation of 5mC oxidation in zygotes resulting from genetic ablation of maternal TET3 is compatible with embryonic development but leads to neonatal sublethality. Our findings thus refine current knowledge of the biological role of 5mC oxidation in zygotes.

## Results

### TET3 mediates 5mC oxidation in both paternal and maternal DNA in zygotes

**Figure 1:**
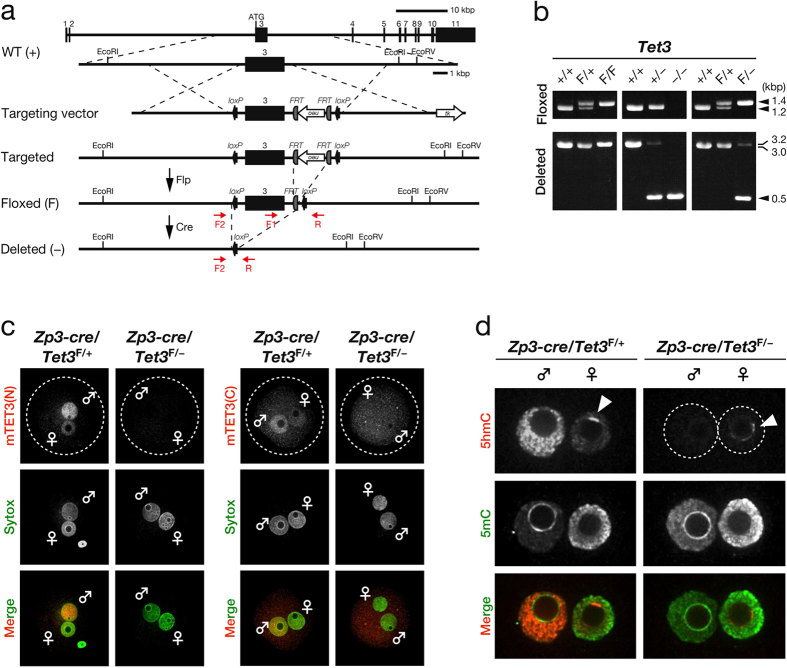
Generation of TET3-depleted oocytes and loss of 5mC oxidation in zygotes derived therefrom. (**a**) Strategy for targeted disruption of mouse *Tet3*. Exons and primers for genotyping are shown as numbered black boxes and red arrows, respectively. See Methods for details. (**b**) Representative genotyping of mice by PCR analysis. The primers F1 and R were used for detection of WT (1.2 kbp) and floxed (1.4 kbp) alleles, whereas primers F2 and R were used for that of WT (3.0 kbp), floxed (3.2 kbp), and deleted (0.5 kbp) alleles. (**c**) Representative images showing depletion of maternal TET3 in zygotes derived from oocytes of [*Zp3-cre*, *Tet3*^F/–^] female mice compared with those derived from oocytes of [*Zp3-cre*, *Tet3*^F/+^] female mice. Zygotes were stained with anti-mTET3(N) or anti-mTET3(C) (red in merged images). Pronuclei were revealed by staining of DNA with Sytox (green in merged images). Dashed circles, ♂, and ♀ indicate the rim of the zygote, the paternal pronucleus, and the maternal pronucleus, respectively. (**d**) Representative images showing the loss of 5hmC in both pronuclei of zygotes derived from oocytes of [*Zp3-cre*, *Tet3*^F/–^] female mice compared with those derived from oocytes of [*Zp3-cre*, *Tet3*^F/+^] female mice. Pronuclei were stained with anti-5hmC (red in merged images) and anti-5mC (green in merged images). ♂, ♀, dashed circles, and arrowheads indicate the paternal pronucleus, the maternal pronucleus, the rim of each pronucleus, and the periphery of the nucleolus-like body, respectively.

To elucidate the biological relevance of TET3-mediated 5mC oxidation in mammalian zygotes, we generated mouse oocytes deficient in TET3. To this end, we first produced mice harboring a “floxed” allele of *Tet3*, in which exon 3 is flanked by *loxP* sites (Fig. 1a). We then crossed these mice with mice harboring a *cre* transgene under the control of the promoter for the zona pellucida glycoprotein 3 gene (*Zp3*). Female mice in which exon 3 of *Tet3* was conditionally ablated as a result of *Zp3* promoter–driven expression of Cre recombinase would be expected to produce oocytes deficient in TET3 (Fig. 1b). To confirm the ablation of TET3 in zygotes derived from oocytes of [*Zp3-cre*, *Tet3*^F/–^] female mice fertilized with wild-type (WT) mouse sperm, we performed immunostaining with antibodies to (anti-) the NH_2_-terminal (N) or COOH-terminal (C) regions of mouse (m) TET3. Signals for both mTET3(N) and mTET3(C) were prominent in the paternal pronucleus of control zygotes derived from oocytes of [*Zp3-cre*, *Tet3*^F/+^] female mice (Fig. 1c), consistent with the previous observation that TET3 predominantly localizes to paternal pronuclei^19^,^33^,^37^. A small amount of mTET3 immunoreactivity was also detected in the maternal pronucleus of such control zygotes (Fig. 1c), however, suggesting that TET3 may also oxidize 5mC in maternal DNA. In contrast, signals for both mTET3(N) and mTET3(C) were virtually absent from both paternal and maternal pronuclei of zygotes derived from oocytes of [*Zp3-cre*, *Tet3*^F/–^] female mice (Fig. 1c). The presence of pronuclei in these zygotes was confirmed by staining of DNA with Sytox (Fig. 1c). These results thus indicated that TET3 in oocytes of [*Zp3-cre*, *Tet3*^F/–^] female mice is depleted efficiently.

Given that TET3 is thought to be responsible for the oxidation of 5mC in zygotes^18^,^19^,^33^, we next investigated the effect of maternal TET3 depletion on this process. Consistent with previous findings^19^,^20^,^21^,^33^,^37^, simultaneous staining of zygotes with anti-5hmC and anti-5mC revealed that the signal for 5mC was prominent in the maternal pronucleus but was virtually absent from the paternal pronucleus of control zygotes derived from oocytes of [*Zp3-cre*, *Tet3*^F/+^] female mice, whereas the opposite pattern was observed for the 5hmC signal (Fig. 1d). Furthermore, loss of the 5hmC signal and an increase in the 5mC signal were apparent in the paternal pronucleus of TET3-deficient zygotes derived from oocytes of [*Zp3-cre*, *Tet3*^F/–^] female mice (Fig. 1d). A small but detectable amount of 5hmC immunoreactivity was observed in the maternal pronucleus of the control zygotes (Fig. 1d), consistent with the low level of TET3 immunoreactivity detected in the maternal pronucleus (Fig. 1c). On the other hand, the signal for 5hmC was virtually undetectable in the maternal pronucleus of TET3-deficient zygotes derived from oocytes of [*Zp3-cre*, *Tet3*^F/–^] female mice, with the exception of staining in a small portion of the periphery of the nucleolus-like body^38^ (Fig. 1d). Together, these results not only verified that maternal TET3 is responsible for the oxidation of paternal 5mC in mouse zygotes, but also suggested that TET3 oxidizes maternal 5mC, albeit to a lesser extent than paternal 5mC.

**Figure 2:**
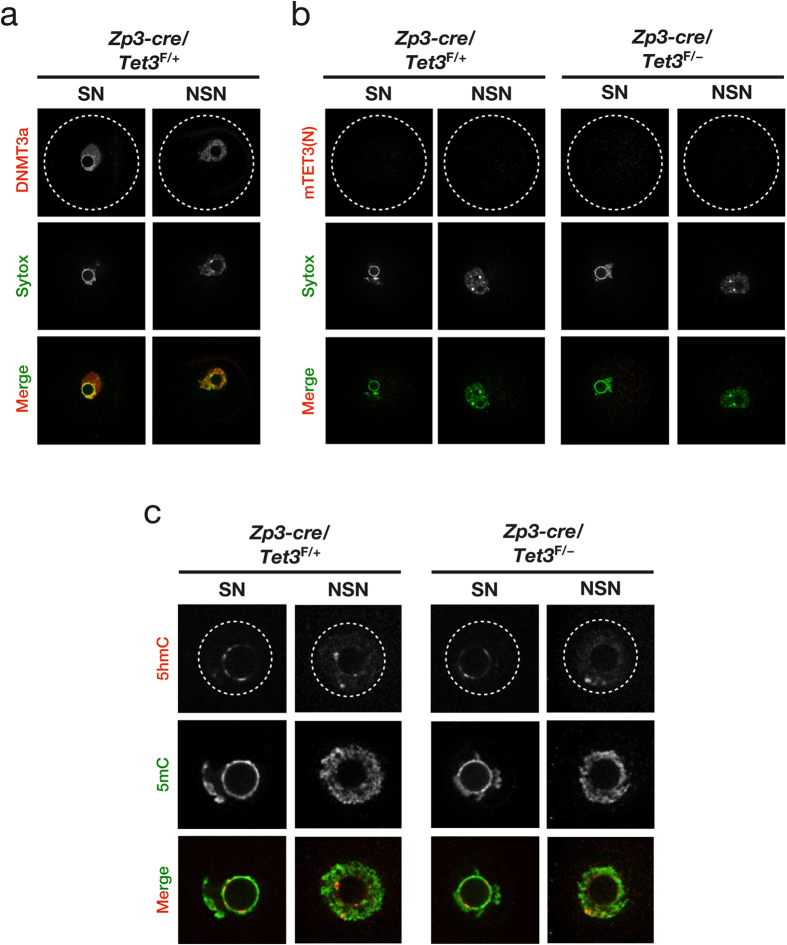
Effect of maternal TET3 depletion on 5mC and 5hmC levels in FG oocytes at the GV stage. (**a**) Representative images showing the accumulation of DNMT3a in both SN and NSN types of nuclei in FG oocytes of [*Zp3-cre*, *Tet3*^F/+^] female mice. Oocytes were stained with anti-DNMT3a (red in merged images), and nuclei were revealed by staining of DNA with Sytox (green in merged images). Dashed circles indicate the rim of the oocyte. (**b**) Representative images showing the absence of maternal TET3 in both SN and NSN types of nuclei in FG oocytes of [*Zp3-cre*, *Tet3*^F/+^] or [*Zp3-cre*, *Tet3*^F/–^] female mice. Oocytes were stained with anti-mTET3(N) (red in merged images), and nuclei were revealed by staining of DNA with Sytox (green in merged images). Dashed circles indicate the rim of the oocyte. (**c**) Representative images showing the presence of 5hmC in both types of nuclei in FG oocytes of both [*Zp3-cre*, *Tet3*^F/–^] and [*Zp3-cre*, *Tet3*^F/+^] female mice. Nuclei were stained with anti-5hmC (red in merged images) and anti-5mC (green in merged images). Dashed circles indicate the rim of the nucleus.

To verify that the signal for 5hmC detected in a small portion of the periphery of the nucleolus-like body of the maternal pronucleus in TET3-deficient zygotes derived from oocytes of [*Zp3-cre*, *Tet3*^F/–^] female mice was not attributable to TET3-mediated oxidation of 5mC, we examined the expression pattern of TET3 and the effect of maternal TET3 depletion on 5mC and 5hmC status in fully grown (FG) oocytes at the germinal vesicle (GV) stage. FG oocytes manifest two distinct types of chromatin configuration: the surrounded nucleolus (SN) type, in which chromatin is highly condensed and surrounds the nucleolus, and the non–surrounded nucleolus (NSN) type, in which chromatin is less condensed and does not surround the nucleolus^39^. DNA methyltransferase 3a (DNMT3a) localizes predominantly to the nucleus of FG oocytes^40^. Immunostaining with anti-DNMT3a confirmed that the DNMT3a signal was prominent in both SN and NSN types of nuclei in control FG oocytes from [*Zp3-cre*, *Tet3*^F/+^] female mice (Fig. 2a). In contrast, signals for both mTET3(N) and mTET3(C) were virtually absent from both SN and NSN types of nuclei in FG oocytes from both [*Zp3-cre*, *Tet3*^F/+^] and [*Zp3-cre*, *Tet3*^F/–^] female mice (Fig. 2b, Supplementary Fig. S1). Simultaneous staining of FG oocytes with anti-5hmC and anti-5mC revealed a small but detectable amount of 5hmC immunoreactivity in both types of nuclei of FG oocytes obtained from both [*Zp3-cre*, *Tet3*^F/+^] and [*Zp3-cre*, *Tet3*^F/–^] female mice (Fig. 2c). In particular, the signal for 5hmC was prominent in a small portion of the periphery of the nucleolus of the SN-type nucleus in FG oocytes, similar to the pattern observed in the maternal pronucleus of zygotes. Furthermore, a reduction in the 5hmC signal or increase in the 5mC signal was not apparent in FG oocytes obtained from [*Zp3-cre*, *Tet3*^F/–^] female mice compared with those from [*Zp3-cre*, *Tet3*^F/+^] females (Fig. 2c), consistent with the expression pattern of TET3 in FG oocytes (Fig. 2b, Supplementary Fig. S1). Together, these results suggested that the 5hmC observed in a small portion of the periphery of the nucleolus-like body of the maternal pronucleus in zygotes is produced during oocyte development before the GV stage and is not generated by TET3-mediated oxidation of 5mC.

### TET3 is dispensable for oocyte development, maturation, and fertilization

**Figure 3:**
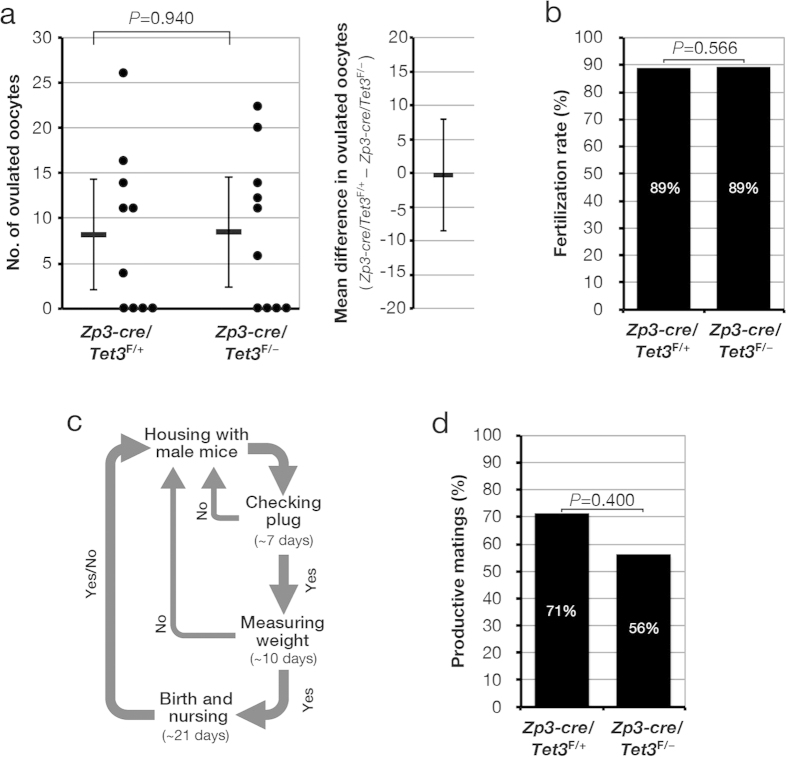
TET3 is dispensable for oocyte development, maturation, and fertilization as well as for pregnancy. (**a**) Number of MII stage oocytes obtained per ovulation for [*Zp3-cre*, *Tet3*^F/+^] (*n* = 10) and [*Zp3-cre*, *Tet3*^F/–^] (*n* = 10) female mice that were treated with PMSG and hCG. The number of ovulated MII stage oocytes for each mouse (circles) as well as the overall mean value (horizontal bar) and 95% confidence interval (CI) are shown in the left panel. The average difference in the number of ovulated MII stage oocytes between [*Zp3-cre*, *Tet3*^F/+^] and [*Zp3-cre*, *Tet3*^*F/*–^] female mice and its 95% CI are indicated in the right panel. Normality and homoscedasticity of the data were verified by the Shapiro-Wilk test (α = 0.05) and F test (α = 0.05), respectively. The *P* value was determined by the two-sided Student’s *t* test. (**b**) Oocytes obtained from [*Zp3-cre*, *Tet3*^F/+^] (*n* = 82) and [*Zp3-cre*, *Tet3*^F/–^] (*n* = 85) mice as in (**a**) were exposed to WT (ICR) spermatozoa *in vitro* for determination of the rate of fertilization, which was judged by the presence of two pronuclei in zygotes. The *P* value was determined by the one-sided Fisher’s exact probability test. (**c**) Strategy for evaluation of productive mating performed over a 2-month observation period. (**d**) [*Zp3-cre*, *Tet3*^F/+^] (*n* = 10) and [*Zp3-cre*, *Tet3*^F/–^] (*n* = 10) female mice were housed with WT (C57BL/6J) male mice as in (**c**) for determination of the number of matings (judged on the basis of plug formation) and the percentage of productive matings (based on the total number of litters produced relative to the total number of matings). The *P* value was determined by the one-sided Fisher’s exact probability test.

Given that we confirmed that TET3 is responsible for 5mC oxidation in zygotes, we evaluated the effect of maternal TET3 depletion on oocyte development, maturation, and fertilization. We collected oocytes at the metaphase II (MII) stage from 8- to 9-week-old [*Zp3-cre*, *Tet3*^F/+^] and [*Zp3-cre*, *Tet3*^F/–^] female mice in which “superovulation” had been induced by injection of pregnant mare serum gonadotropin (PMSG) and human chorionic gonadotropin (hCG). The oocytes were then fertilized with WT sperm *in vitro*. Consistent with previous observations^19^, the number of ovulated oocytes obtained from [*Zp3-cre*, *Tet3*^F/–^] female mice and their fertilization rate did not differ significantly from those for [*Zp3-cre*, *Tet3*^F/+^] female mice (Fig. 3a,b; Supplementary Table S1), suggesting that TET3-mediated 5mC oxidation is dispensable for oocyte development after the primary follicle stage (at which expression of the *cre* transgene is initiated as a result of activation of the *Zp3* promoter) as well as for oocyte maturation and fertilization.

We next determined whether maternal TET3 deficiency affects pregnancy rate. [*Zp3-cre*, *Tet3*^F/+^] or [*Zp3-cre*, *Tet3*^F/–^] female mice were housed with WT males, and the pregnancy rate was evaluated over a 2-month observation period (Fig. 3c). The pregnancy rate was determined as successful pregnancy per mating and did not differ significantly between [*Zp3-cre*, *Tet3*^F/+^] female mice (71%) and [*Zp3-cre*, *Tet3*^F/–^] female mice (56%) (Fig. 3d, Supplementary Table S2). Although this finding is not consistent with previous results obtained with [*Tnap-cre*, *Tet3*^F/+^] and [*Tnap-cre*, *Tet3*^F/–^] female mice, which had pregnancy rates of 96.3% and 43.8%, respectively (*P* < 0.0001)^19^, the discrepancy might be explained by differences in the experimental conditions including the genetic background of the mice, the deleted region of *Tet3*, and the promoter regulating the expression of *cre*. Our results thus suggested that TET3-mediated 5mC oxidation in zygotes is not essential for pregnancy.

### TET3-mediated 5mC oxidation in zygotes is not essential for mouse development but is required for neonatal growth

**Figure 4:**
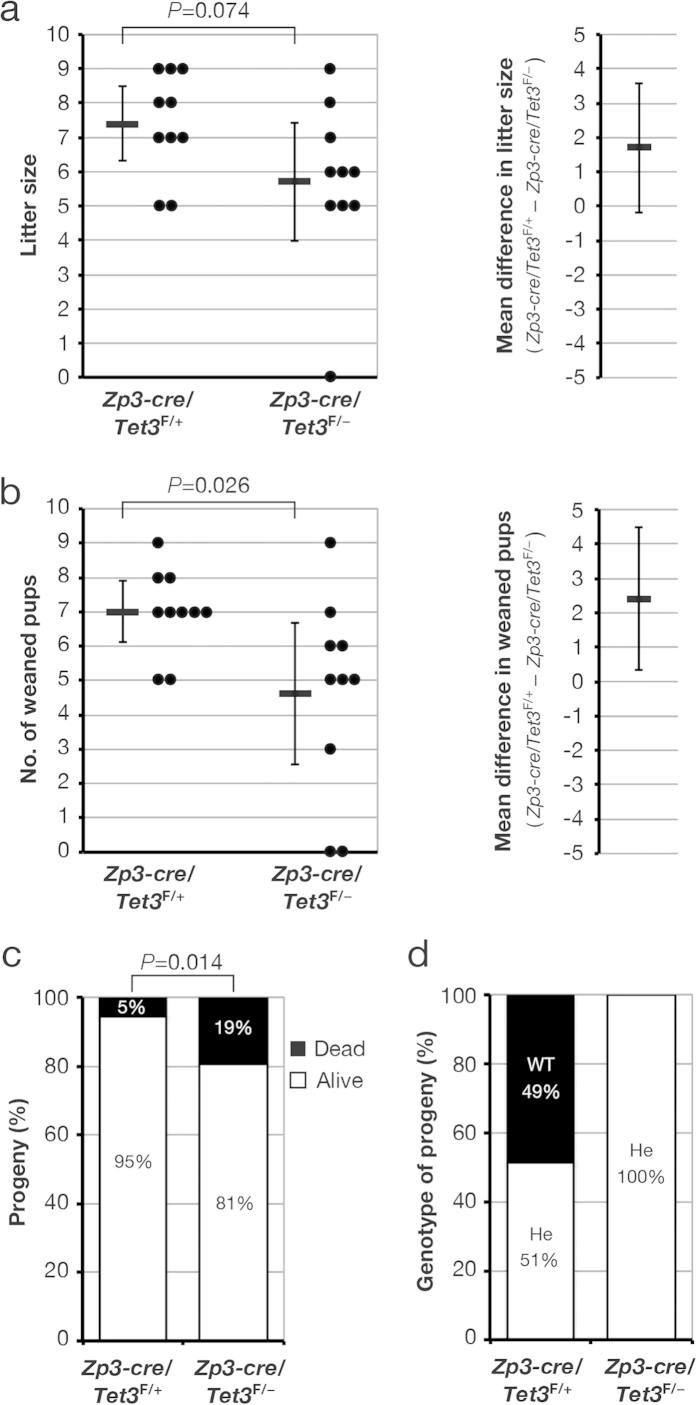
TET3-mediated 5mC oxidation in the zygote is not required for mouse development. (**a,b**) [*Zp3-cre*, *Tet3*^F/+^] (*n* = 10) or [*Zp3-cre*, *Tet3*^F/–^] (*n* = 10) female mice were housed with WT (C57BL/6J) males as in Fig. 3c, and both litter size (**a**) and the number of weaned pups (**b**) were determined. Litter size and the number of weaned pups for each female (circles) as well as the overall mean values (horizontal bars) and 95% CIs are shown in the left panels, and the average difference in litter size or the number of weaned pups between [*Zp3-cre*, *Tet3*^F/+^] and [*Zp3-cre*, *Tet3*^F/–^] dams and its 95% CI are indicated in the right panels. Normality and homoscedasticity of the data were verified by the Shapiro-Wilk test (α = 0.05) and F test (α = 0.05), respectively. The *P* values were determined by the two-sided Student’s *t* test. (**c**) The offspring produced in (**a**) ([*Zp3-cre*, *Tet3*^F/+^] dams, *n* = 74; [*Zp3-cre*, *Tet3*^F/–^] dams, *n* = 57) were counted at 7 days after birth, and the percentages of live and dead pups were determined. The *P* value was determined by the one-sided Fisher’s exact probability test. (**d**) *Tet3* genotype of weaned mice in (**b**) ([*Zp3-cre*, *Tet3*^F/+^] dams, *n* = 70; [*Zp3-cre*, *Tet3*^F/–^] dams, *n* = 46). He, heterozygous.

Given that we found that TET3 is dispensable for oocyte development, maturation, and fertilization, we evaluated the effect of maternal TET3 deficiency on fecundity. [*Zp3-cre*, *Tet3*^F/+^] or [*Zp3-cre*, *Tet3*^F/–^] female mice were housed with WT males and were assessed for fecundity (litter size) over a 2-month period as indicated in Fig. 3c. The mean litter sizes of [*Zp3-cre*, *Tet3*^F/+^] and [*Zp3-cre*, *Tet3*^F/–^] females were 7.4 and 5.7 (23% reduction, *P* = 0.074), respectively (Fig. 4a, Supplementary Table S3), suggesting that TET3 deficiency tends to reduce litter size, possibly as a result of increased developmental failure. Although the effect of maternal TET3 deficiency on litter size was not as marked as that described previously for [*Tnap-cre*, *Tet3*^F/–^] female mice (litter size of 2.8, compared with a value of 8.4 for [*Tnap-cre*, *Tet3*^F/+^] females; 67% reduction, *P* < 0.0001)^19^, this discrepancy again might be attributable to differences in experimental conditions between the two studies. Our results thus suggested that attenuation of zygotic 5mC oxidation is compatible with mouse development.

We next assessed the effect of maternal TET3 deficiency on neonatal and postnatal growth by examining the progeny of [*Zp3-cre*, *Tet3*^F/+^] and [*Zp3-cre*, *Tet3*^F/–^] female mice mated with WT males. Offspring were nursed for 21 days after birth and then weaned (Fig. 3c). The mean number of weaned pups for [*Zp3-cre*, *Tet3*^F/–^] dams was significantly reduced compared with that for [*Zp3-cre*, *Tet3*^F/+^] dams (4.6 versus 7.0) (Fig. 4b, Supplementary Table S3), whereas the percentage of dead pups was significantly greater for [*Zp3-cre*, *Tet3*^F/–^] female mice (19.3%) than for [*Zp3-cre*, *Tet3*^F/+^] female mice (5.4%) (Fig. 4c). All pups that were alive at 7 days after birth survived and were weaned at 21 days after birth. We confirmed that all of the weaned pups of [*Zp3-cre*, *Tet3*^F/–^] female mice and about half of those of [*Zp3-cre*, *Tet3*^F/+^] female mice were heterozygous for *Tet3* (Fig. 4d, Supplementary Table S4), indicating that the floxed *Tet3* allele is efficiently ablated by Cre recombinase produced from *Zp3-cre*. These results thus suggested that deficiency of maternal TET3 leads to neonatal sublethality.

### Maternal TET3 contributes to the fine-tuning of zygotic transcription after DNA synthesis

**Figure 5:**
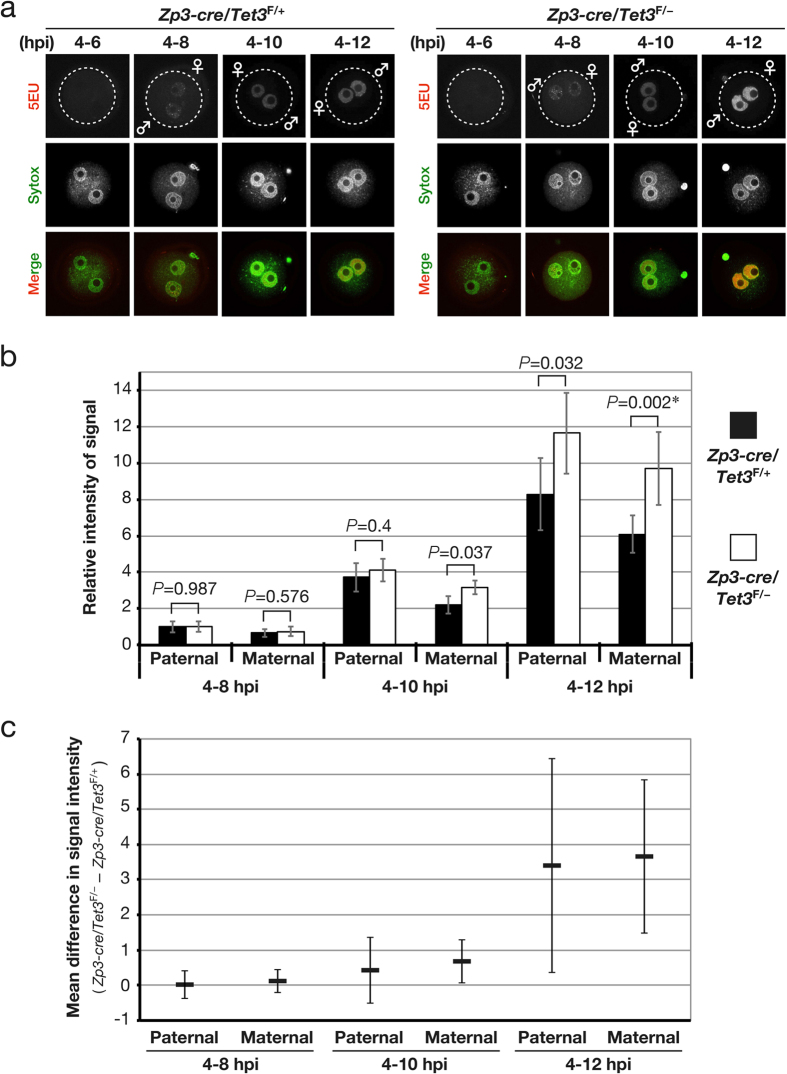
Depletion of maternal TET3 in zygotes enhances zygotic transcription. (**a**) Representative images showing accumulation of incorporated 5EU in zygotes derived from oocytes of [*Zp3-cre*, *Tet3*^F/+^] or [*Zp3-cre*, *Tet3*^F/–^] female mice. Zygotes were pulse-labeled with 5EU from 4 to 6, 8, 10, or 12 hpi, incorporated 5EU was then observed (red in merged images). Pronuclei were revealed by staining of DNA with Sytox (green in merged images). Dashed circles, ♂, and ♀ indicate the rim of the zygote, the paternal pronucleus, and the maternal pronucleus, respectively. (**b,c**) The signal intensity for incorporated 5EU in the paternal and maternal pronuclei of zygotes derived from oocytes of [*Zp3-cre*, *Tet3*^F/–^] (4–8 hpi, *n* = 11; 4–10 hpi, *n* = 15; 4–12 hpi, *n* = 15) or [*Zp3-cre*, *Tet3*^F/+^] (4–8 hpi, *n* = 9; 4–10 hpi, *n* = 11; 4–12 hpi, *n* = 10) female mice was quantified, normalized by that of Sytox, and expressed relative to the normalized value for the paternal pronucleus of zygotes (4–8 hpi) derived from oocytes of [*Zp3-cre*, *Tet3*^F/+^] females. The overall mean values and 95% CIs are shown in (**b**), and the average difference (and its 95% CI) in the normalized values between zygotes derived from oocytes of [*Zp3-cre*, *Tet3*^F/–^] female mice and those derived from oocytes of [*Zp3-cre*, *Tet3*^F/+^] females is indicated in (**c**). Normality and homoscedasticity of the data were verified by the Shapiro-Wilk test (α = 0.05) and F test (α = 0.05), respectively. The *P* values were determined by the two-sided Student’s *t* test, with the exception of that indicated by the asterisk, which was determined by the two-sided Welch’s *t* test because of unequal sample variance.

Given that the onset of zygotic transcription as well as that of oxidation of 5mC occurs shortly after pronucleus formation in zygotes, we investigated the effect of maternal TET3 depletion on zygotic transcription. To this end, we analyzed RNA synthesis by measuring the incorporation of 5-ethynyl uridine (5EU) into newly synthesized RNA. The signal for 5EU was detected in zygotes derived from oocytes of both [*Zp3-cre*, *Tet3*^F/+^] and [*Zp3-cre*, *Tet3*^F/–^] female mice as early as 6 to 8 h postinsemination (hpi), but it was not detected before 6 hpi (Fig. 5a,b). Although transcribed RNA accumulated to similar extents in zygotes derived from oocytes of both [*Zp3-cre*, *Tet3*^F/+^] and [*Zp3-cre*, *Tet3*^F/–^] female mice until 10 hpi, the amount of transcribed RNA was significantly increased in both maternal and paternal pronuclei of zygotes derived from oocytes of [*Zp3-cre*, *Tet3*^F/–^] females after 10 hpi (Fig. 5). Zygotes treated with α-amanitin, an inhibitor of RNA polumerase II, did not yield a signal for 5EU (Supplementary Fig. S2), indicating that the detected 5EU signals were indeed attributable to zygotic transcribed RNA. These results thus suggested that maternal TET3 or its oxidation of 5mC restrains zygotic transcription.

To exclude the possibility that the observed effect of maternal TET3 depletion on zygotic transcription might be attributable to a difference in cell cycle progression between zygotes derived from oocytes of [*Zp3-cre*, *Tet3*^F/+^] or [*Zp3-cre*, *Tet3*^F/–^] female mice, we examined the effect of maternal TET3 depletion on the cell cycle of zygotes. We analyzed DNA synthesis by measurement of the incorporation of 5-ethynyl-2′-deoxyuridine (5EdU) into newly synthesized DNA. A homogeneous signal for 5EdU in both pronuclei, a feature of the early replication stage^41^, was detected in ~90% of zygotes derived from oocytes of both [*Zp3-cre*, *Tet3*^F/+^] and [*Zp3-cre*, *Tet3*^F/–^] female mice as early as 4 to 6 hpi, indicating that most zygotes entered S phase of the cell cycle (Supplementary Fig. S3a,b). A signal for 5EdU at the periphery of the nucleolus-like body in the maternal pronucleus and at the periphery of the paternal pronucleus, a feature of late replication^41^, was detected in 27% of zygotes derived from oocytes of both [*Zp3-cre*, *Tet3*^F/+^] and [*Zp3-cre*, *Tet3*^F/–^] female mice, whereas a signal was not detected in the remaining zygotes, at 9 to 10 hpi (Supplementary Fig. S3a,b), indicating that >70% of zygotes had exited S phase of the cell cycle. Zygotes treated with aphidicolin, an inhibitor of B-family DNA polymerases, did not yield a signal for 5EdU (Supplementary Fig. S3c), indicating that the signals attributed to newly synthesized DNA were specific. These results suggested that maternal TET3 depletion has no obvious effect on cell cycle progression but enhances zygotic transcription after completion of DNA replication.

## Discussion

We have here shown that the loss of maternal TET3 results in attenuation of 5mC oxidation in mouse zygotes and neonatal sublethality. Our genetic evidence suggests that TET3 oxidizes not only paternal 5mC but also, albeit to a lesser extent, maternal 5mC, consistent with the relative extent of its localization to the two pronuclei of zygotes. Our data further indicate that TET3-mediated oxidation of paternal and maternal 5mC in zygotes is not essential for mouse development but is required for neonatal growth.

It has been thought that paternal DNA is subject to a genome-wide loss of 5mC, whereas 5mC of maternal DNA is resistant to this process. This notion was based on the initial observation that genome-wide loss of 5mC occurs asymmetrically in mouse zygotes^2^,^3^. Consistent with this notion, only paternal 5mC was found to be oxidized concomitantly with the loss of 5mC^19^,^20^,^21^,^33^,^37^, and TET3, which is responsible for the oxidation of 5mC in zygotes, was found to localize specifically to the paternal pronuclus^19^,^33^,^37^. Recent studies indicate that TET3 activity is regulated by a cullin–RING finger ligase–4 complex^42^,^43^. However, other studies have found no dynamic reciprocal changes in the levels of 5mC and its oxidation product^41^,^44^. We have now shown that TET3 is present not only in the paternal pronucleus but also in the maternal pronucleus of mouse zygotes. Consistent with the presence of TET3 in both pronuclei, oxidation of 5mC to 5hmC was detected in both paternal and maternal pronuclei. However, the oxidation of maternal 5mC was less pronounced compared with that of paternal 5mC, corresponding to the lesser extent of TET3 localization to the maternal pronucleus than to the paternal pronucleus. Furthermore, other groups have recently described similar observations^34^,^36^. Our data coupled with these previous observations support the notion that not only paternal DNA but also maternal DNA in zygotes is subject to TET3-mediated oxidation of 5mC, although such oxidation occurs to a lesser extent in maternal DNA.

Pericentric heterochromatin is arranged in the region surrounding the nucleolus in FG oocytes^45^. This organization of pericentric heterochromatin is reiterated later in the zygote^46^ and correlates with the developmental competence of the embryo^47^,^48^. Similar to previous observations^18^,^41^, we found that 5hmC accumulates in a region of the periphery around the nucleolus-like body in the maternal pronucleus of zygotes. However, this accumulation of 5hmC was not completely abrogated in zygotes deficient in TET3, in contrast to the complete loss of 5hmC from the nucleoplasm apparent in TET3-depleted zygotes. We also detected TET3-independent generation of 5hmC in both SN and NSN types of FG oocytes at the GV stage. Given that 5hmC is enriched in a subset of pericentric heterochromatin of primordial germ cells (PGCs) and that this pattern of 5hmC subcellular localization is maintained even in mature oocytes^49^, it is likely that the accumulation of 5hmC in a region of the periphery around the nucleolus-like body in the maternal pronucleus of zygotes is established in PGCs and corresponds to pericentric heterochromatin of a subset of chromosomes. Pericentric 5hmC in PGCs was recently suggested to be generated by TET1^50^. Although the meiotic phenotype of *Tet1* knockout female PGCs suggests that TET1 may have an important role in germ cell development^50^, its roles in the zygote as well as in embryonic development are unclear. Whether pericentric 5hmC in the maternal pronucleus of zygotes is important for embryonic development warrants further investigation.

A genome-wide loss of 5mC in the paternal pronucleus of zygotes has been thought to play an important role in mammalian development. Consistent with this notion, TET3-mediated oxidation of paternal 5mC has also been thought to be required for mouse development^51^,^52^,^53^. However, our genetic evidence now suggests that TET3-mediated oxidation of paternal 5mC in zygotes is not essential for mouse development. We have shown that TET3 is dispensable for oocyte development, maturation, and fertilization, consistent with the results of a previous study^19^. Deficiency of TET3 and the accompanying attenuation of 5mC oxidation in zygotes appeared to result in a small but nonsignificant increase in developmental failure of embryos (23% reduction in litter size, *P* = 0.074). However, this result contrasts with the pronounced developmental failure of embryos with maternal TET3 deficiency observed in a previous study (67% reduction in litter size, *P* < 0.0001)^19^. This phenotypic difference may be attributable to differences in experimental conditions between the two studies. First, we used the *Zp3* promoter to control expression of *cre* and thereby to achieve oocyte-specific deletion of *Tet3* from the primary follicle stage, whereas the *Tnap* promoter was used in the previous study to achieve germline-specific deletion of *Tet3* in PGCs. Ablation of *Tet3* in different cell types as well as at different developmental stages might lead to distinct phenotypes. Second, we deleted exon 3 of *Tet3*, whereas exons 8 and 9 were eliminated in the previous study^19^. The deleted genomic regions might contain functional elements such as enhancers or produce noncoding RNAs, and their loss might therefore have phenotypic consequences. Third, we studied mice with a C57BL/6 genetic background, whereas the mice in the previous study had a mixed C57BL/6–129Sv genetic background^19^. Genetic background might have a substantial effect on the phenotype associated with loss of chromatin modifiers. For example, deficiency of the histone demethylase Jumonji domain–containing protein 1A (JMJD1A, also known as KDM3A), which controls *Sry* expression by regulating H3K9me2 (dimethylation at Lys^9^ of histone H3) marks, results in a male-to-female sex-reversal phenotype^54^. However, this phenotype is dependent not only on the loss of *Jmjd1a* but also on the genetic origin of the Y chromosome. The proportion of JMJD1A-deficient mice that manifested abnormal sex differentiation was thus 88% with a Y chromosome of CBA origin but only 14% with one of C57BL/6J origin, even though the H3K9me2 levels at the *Sry* locus in the JMJD1A-deficient gonads at embryonic day 11.5 were indistinguishable between these genetic backgrounds. During the revision of our manuscript, another group using complementary methods independently found that TET3-mediated oxidation of paternal 5mC is dispensable for mouse development^55^. Our data coupled with this new study thus strongly support the notion that oxidation of 5mC mediated by maternal TET3 in zygotes is not essential for mouse development.

Recent genome-scale sequence analysis revealed that a much smaller proportion of 5mC is oxidized in the paternal genome of zygotes than previously thought^34^,^36^. In addition, oxidized 5mC was found to be diluted in a manner dependent on DNA replication^20^,^21^. These findings indicate that the oxidation of 5mC contributes to DNA demethylation in zygotes to a lesser extent than previously thought and are therefore consistent with our results showing that deficiency of TET3 and consequent attenuation of 5mC oxidation in zygotes are compatible with mouse development. This compatibility might be attributable to compensation for TET3 deficiency by replication-dependent successive dilution of 5mC as well as by oxidation of 5mC mediated by TET1 or TET2 during preimplantation development. Although TET3-mediated 5mC oxidation is not essential for embryonic development, we found that maternal TET3 is required for neonatal growth. Given that *Tet3* homozygous mutant mice manifest neonatal lethality, the observed neonatal sublethality of *Tet3* heterozygous progeny of [*Zp3-cre*, *Tet3*^F/–^] female mice might be expected to be attributable to the haploinsufficiency of *Tet3*. [*Zp3-cre*, *Tet3*^F/+^] female mice mated with WT males also generate *Tet3* heterozygous progeny at a theoretical proportion of 50%. If these latter pups exhibit neonatal sublethality due to haploinsufficiency of *Tet3*, then this predicted ratio would be affected. However, the ratio of *Tet3* genotypes for the weaned progeny matched the theoretical one, eliminating the possibility that haploinsufficiency of *Tet3* has a sublethal effect. During the revision of our manuscript, another group using complementary methods independently found that deficiency of maternal TET3 leads to neonatal sublethality^55^. Our data coupled with this new study thus strongly support the notion that TET3 is required for neonatal growth.

The oocyte-to-embryo transition is accompanied by a marked reprogramming of gene expression. Mammalian FG oocytes are transcriptionally quiescent, with oocytes after this stage as well as zygotes utilizing only transcripts synthesized and stored during early oocyte development until zygotic genome activation. The timing of zygotic genome activation is species dependent, and the onset of zygotic transcription as well as that of oxidation of 5mC occurs shortly after pronucleus formation in mouse zygotes. Our genetic evidence now suggests that TET3 or its oxidation of 5mC in zygotes represses zygotic transcription. We found that depletion of maternal TET3 did not influence cell cycle progression, however. Deficiency of TET3 and the associated attenuation of 5mC oxidation in zygotes thus appear to result in an increase in zygotic transcription after DNA replication. Although RNA interference–mediated depletion of maternal TET3 was previously shown to have no apparent effect on zygotic transcription (as determined by measurement of 5-bromouridine-5′-triphosphate incorporation into permeabilized zygotes over 15 min)^33^, our measurement of 5EU incorporation into intact living zygotes over 2 to 8 h revealed that maternal TET3 may function as a transcriptional repressor in zygotes. TET proteins have been expected to regulate transcription by adjusting the level of DNA methylation at promoters^55^. However, the extent of transcriptional activation by TET1 was found to be less pronounced than its observed repressive function mediated through the recruitment of polycomb repressive complex 2 and the Sin3A corepressor complex^56^. The repressive effect of TET3 on zygotic transcription observed in the present study might thus be explained by a mechanism similar to that described for TET1. Given that epigenetic mutations tend to have a milder phenotype compared with genetic mutations, the accumulation of moderate effects of maternal TET3 depletion each occurring during a narrow time window, such as the effect on zygotic transcription described here, may give rise to the neonatal sublethality apparent in *Tet3* heterozygous progeny of [*Zp3-cre*, *Tet3*^F/–^] female mice. Further studies are thus required to determine why TET3 is required for neonatal growth.

## Methods

### Generation of *Tet3* conditional knockout mice

To generate *Tet3* conditional knockout mice, we inserted two *loxP* sequences into the *Tet3* locus by homologous recombination. For construction of the targeting vector, a 6.3-kbp mouse genomic fragment of the 5′ proximal region of *Tet3*, a 2.4-kbp fragment containing exon 3, and a 5.5-kbp fragment containing intron 3 (Gene ID, 194388; RefSeq, NM_183138) were amplified by the polymerase chain reaction (PCR) from genomic DNA of RENKA embryonic stem (ES) cells (C57BL/6N)^57^. The 2.4-kbp amplified fragment containing exon 3 was cloned between the two *loxP* sites of a vector containing both a PGK_neo cassette (neomycin resistance gene driven by the phosphoglycerate kinase 1 gene promoter) as a positive selection marker flanked by *FRT* sequences as well as a PGK_TK cassette (thymidine kinase gene driven by the phosphoglycerate kinase 1 gene promoter) as a negative selection marker. The 6.3- and 5.5-kbp fragments were then also subcloned into the resulting plasmid. The resulting targeting vector contains a 5′ homologous region of 6.3 kbp, the first *loxP* site, the 2.4-kbp floxed genomic region containing exon 3, the PGK_neo cassette flanked by *FRT* sites, the second *loxP* site, a 3′ homologous region of 5.5 kbp, and the PGK_TK cassette (Fig. 1a). The targeting vector was linearized and introduced into RENKA ES cells by electroporation. The cells were subjected to selection with geneticin and ganciclovir, resistant clones were isolated, and their DNA was screened for homologous recombination by nested-PCR analysis with the primers *3AR4* (5′-TCACCTGGTCTATACATCTGAATGC-3′) and *neo_108r* (5′-CCTCAGAAGAACTCGTCAAGAAG-3′) for the first PCR and *3AR3* (5′-CAGTCACTCAATATTTCAACACTCC-3′) and *neo_100* (5′-AGGTGAGATGACAGGAGATC-3′) for the nested PCR. PCR-positive ES clones were expanded, and their isolated DNA was further analyzed by PCR with the primers *5AF3* (5′-AAGTCTCAAACTCTTCTCTGTGTCC-3′) and *neo_marker_sense* (5′-ATTCGCAGCGCATCGCCTTCTATCGCCTTC-3′) for amplification of the 5′ region, *3AR3* and *neo_100* for amplification of the 3′ region, and *DAF2* (5′-AAAGTCGACCCAGCTAGAAGGTGTAGACTGAGGC-3′) and *R65462* (5′-CGTAAGATGACACAGCTTCG-3′) for amplification of the first *loxP* region. Homologous recombination in these clones was also confirmed by genomic Southern hybridization with a probe for the neomycin resistance gene.

Homologous recombinant ES cell clones were aggregated with ICR eight-cell embryos to generate chimeric mice. Chimeric mice with a high contribution of the RENKA background were crossed with C57BL/6N mice to obtain offspring heterozygous for the modified *Tet3* allele. The targeted allele was identified by PCR analysis with the primer sets *3AR3* and *neo_100* as well as *DAF2* and *R65462.* The positive selection marker (PGK_neo cassette) was removed from the genome by crossing the heterozygous mice with C57BL/6J mice harboring a *Flp* transgene. The resulting offspring were then crossed with mice harboring a *cre* transgene under the control of the *Zp3* promoter^58^. For genotyping of mice, DNA was extracted from embryos or the tail tip and was subjected to PCR analysis with the primers F1 (5′-TGTTAGGCAGATTGTTCTGG-3′) and R (5′-GAAGGCCTCTGTGATGTCAG-3′) for detection of the floxed allele as well as F2 (5′-GGAGAACCTGCAAGGAAAGCG-3′) and R for detection of the deleted allele.

### Generation of antibodies to TET3

Plasmids encoding glutathione S-transferase (GST) fusion proteins of mTET3 were constructed by PCR amplification with cDNA prepared from NIH 3T3 cells. Plasmids encoding GST-mTET3(N) (RefSeq NP_898961, amino acids 1–200) or GST-mTET3(C) (RefSeq NP_898961, amino acids 1300–1506) were generated by insertion of the coding sequence into the SmaI and HindIII sites of a modified pGEX-2T vector (GE Healthcare Bio-Sciences). All constructs were verified by sequencing. The GST-mTET3 fusion proteins were purified as previously described^59^. Polyclonal antibodies to mTET3(N) and to mTET3(C) were generated by injection of rabbits with the corresponding recombinant proteins and were then purified as previously described^60^.

### Isolation of spermatozoa and oocytes as well as *in vitro* fertilization

All mice were maintained under the specific pathogen–free condition, and all animal experiments were approved by the animal care and use committee of Kyushu University and were performed in accordance with the regulations for animal experiments at Kyushu University. FG oocytes at the GV stage were obtained from 8- to 9-week-old [*Zp3-cre*, *Tet3*^F/–^] or [*Zp3-cre*, *Tet3*^F/+^] female mice that had been treated with PMSG (ASKA Pharmaceutical Co.). The ovaries were removed from the mice and transferred to modified human tubal fluid (HTF) medium (Irvine Scientific). The ovarian follicles were punctured with a 27-gauge needle, and the cumulus cells were gently removed from the cumulus-oocyte complexes with the use of a narrow-bore glass pipette. MII stage oocytes were collected from the oviducts of 8- to 9-week-old [*Zp3-cre*, *Tet3*^F/–^] or [*Zp3-cre*, *Tet3*^F/+^] female mice that had been treated with PMSG and hCG (ASKA Pharmaceutical Co.), and spermatozoa were collected from the cauda epididymis of 10- to 15-week-old ICR or C57BL/6J male mice (CLEA Japan). For *in vitro* fertilization, MII stage oocytes were placed in HTF medium supplemented with bovine serum albumin (BSA) at 10 mg/ml (Sigma-Aldrich) and were exposed to spermatozoa in which capacitation had been induced by prior incubation for 1.5 h in HTF medium supplemented with BSA at 4 mg/ml. At 5 h after insemination, fertilized oocytes with two pronuclei were washed and cultured under a humidified atmosphere of 5% CO_2_/5% O_2_/90% N_2_ at 37 °C in HTF medium supplemented with BSA (4 mg/ml). The fertilization rate of oocytes was judged on the basis of the presence of two pronuclei in zygotes at 8 h after insemination.

### Immunostaining of oocytes and zygotes

FG oocytes at the GV stage and zygotes produced by *in vitro* fertilization were fixed for 20 min at room temperature with 4% paraformaldehyde in phosphate-buffered saline (PBS) containing 0.2% Triton X-100. They were then washed with PBS containing 0.05% Tween 20 (PBST) before incubation first for 1 h at room temperature with 1% BSA in PBST and then overnight at 4 °C with rabbit anti-mTET3(N), anti-mTET3(C), or anti-DNMT3a (Imgenex) (each at a 1:1000 dilution). They were then washed with PBST, incubated for 1 h at room temperature with Alexa Fluor 568–conjugated goat antibodies to rabbit immunoglobulin G (Molecular Probes–Invitrogen), washed with PBST, and mounted in SlowFade Gold antifade reagent (Molecular Probes–Invitrogen) containing Sytox (Molecular Probes–Invitrogen). For immunostaining of 5hmC and 5mC, FG oocytes at the GV stage and zygotes were fixed with 4% paraformaldehyde in PBS for 30 min at room temperature and then permeabilized with 0.4% Triton X-100 for 30 min at room temperature. They were then washed with PBST, treated with 4 M HCl for 10 min at room temperature, neutralized with 0.1 M Tris-HCl (pH 8.5) for 10 min, and washed with PBST containing 0.5 M NaCl. For the following steps, all solutions contained 0.5 M NaCl. The oocytes and zygotes were incubated first for 1 h at room temperature with 1% BSA in PBST and then overnight at 4 °C with rabbit anti-5hmC (1:1000 dilution, Active Motif) and mouse anti-5mC (1:100, Eurogentec). They were then washed with PBST, incubated for 1 h at room temperature with Alexa Fluor 488–conjugated goat antibodies to mouse immunoglobulin G and Alexa Fluor 568–conjugated goat antibodies to rabbit immunoglobulin G (Molecular Probes–Invitrogen), washed with PBST, and mounted in SlowFade Gold antifade reagent. Fluorescence images were acquired at multiple 2-μm intervals in the *z*-axis with the use of a confocal microscope (CV1000, Yokogawa).

### 5EU and 5EdU incorporation assays

Incorporation of 5EU and 5EdU in zygotes was monitored on the basis of their fluorescence labels with the use of a Click-iT RNA Imaging Kit and a Click-iT EdU Imaging Kit (Life Technologies), respectively. The zygotes were mounted in SlowFade Gold antifade reagent containing Sytox. Fluorescence images were acquired at multiple 2-μm intervals in the *z*-axis with the use of a confocal microscope (CV1000, Yokogawa), and the intensity of fluorescence signals for incorporated 5EU and for Sytox was quantified with the use of MetaMorph software (Universal Imaging Co.).

### Fecundity test

Eight-week-old [*Zp3-cre*, *Tet3*^F/–^] or [*Zp3-cre*, *Tet3*^F/+^] female mice were housed with 8-week-old WT (C57BL/6J) male mice, and a check for plug formation was performed daily each morning for 7 days. If no plug was detected, the male mouse was replaced with another one. The weight of female mice was measured at 10 days after plug formation. If no increase in weight was observed, the female mouse was housed with another WT male. Dams were allowed to nurse their pups for 21 days after delivery and were then again housed with a WT male. Weaned pups were counted at 21 days after birth. This fecundity test was conducted over a 2-month observation period.

### Statistical analysis

Data are presented and were analyzed as described in legends. A *P* value of <0.05 was considered statistically significant.

## Additional Information

**How to cite this article**: Tsukada, Y. *et al.* Maternal TET3 is dispensable for embryonic development but is required for neonatal growth. *Sci. Rep.* **5**, 15876; doi: 10.1038/srep15876 (2015).

## Supplementary Material

Supplementary Information
