# Supplementary material for: Maternal TET3 is dispensable for embryonic development but is required for neonatal growth
Source: Sci Rep. 2015 Oct 28;5:15876. doi: 10.1038/srep15876 (PMC4623673; doi:10.1038/srep15876)

Supplementary Information

Maternal TET3 is dispensable for embryonic development but is required for neonatal growth

Yu-ichi Tsukada*, Tomohiko Akiyama, Keiichi I. Nakayama

*Corresponding author

Yu-ichi Tsukada: ytsukada@ifrc.kyushu-u.ac.jp

Supplementary Tables S1–S4

Supplementary Figure Legends S1–S3

Supplementary Figures S1–S3

Supplementary Table S1. Development, maturation, and fertilization of oocytes derived from [*Zp3-cre*, *Tet3*$^{F/+}$] or [*Zp3-cre*, *Tet3*$^{F/–}$] female mice.

Female genotype	No. of females	Total no. of ovulated oocytes	Mean no. of ovulated oocytes/female (95% CI)	Total no. of oocytes fertilized	Fertilization rate (%)
Zp3-cre, *Tet3*$^{F/+}$	10	82	8.2 (1.8–14.6)	73	89
Zp3-cre, *Tet3*$^{F/–}$	10	85	8.5 (2.4–14.6)	76	89

Data correspond to those in Figure 3a, b.

Supplementary Table S2. Productive mating of [*Zp3-cre*, *Tet3*$^{F/+}$] or [*Zp3-cre*, *Tet3*$^{F/–}$] female mice.

Female genotype	No. of females	Total no. of matings	Total no. of litters	Productive matings (%)
Zp3-cre, *Tet3*$^{F/+}$	10	14	10	71
Zp3-cre, *Tet3*$^{F/–}$	10	16	9	56

Data correspond to those in Figure 3d.

Supplementary Table S3. Litter size and neonatal growth of the offspring of [*Zp3-cre*, *Tet3*$^{F/+}$] or [*Zp3-cre*, *Tet3*$^{F/–}$] female mice.

Female genotype	No. of females	Total no. of viable pups born	Mean litter size (95% CI)	Total no. of weaned pups	Mean no. of weaned pups (95% CI)
Zp3-cre, *Tet3*$^{F/+}$	10	74	7.4 (6.3–8.5)	70	7.0 (6.1–7.9)
Zp3-cre, *Tet3*$^{F/–}$	10	57	5.7 (4.0–7.4)	46	4.6 (2.5–6.7)

Data correspond to those in Figure 4a, b.

Supplementary Table S4. ***Tet3*** **genotype of weaned progeny of [*Zp3-cre*, *Tet3*$^{F/+}$] or [*Zp3-cre*, *Tet3*$^{F/–}$] female mice.**

Female genotype	Genotype of progeny		Total no. of weaned pups
	(+/+)	(+/–)	
Zp3-cre, *Tet3*$^{F/+}$	34 (49%) [19 ♀ (56%), 15 ♂ (44%)]	36 (51%) [20 ♀ (56%), 16 ♂ (44%)]	70
Zp3-cre, *Tet3*$^{F/–}$	0 (0%)	46 (100%) [22 ♀ (48%), 24 ♂ (52%)]	46

Data correspond to those in Figure 4d.

Supplementary Figure Legends

Supplementary Figure S1. TET3 is not enriched in the nucleus of FG oocytes.

Representative images showing the absence of maternal TET3 in SN or NSN types of nuclei in FG oocytes of [*Zp3-cre*, *Tet3*$^{F/+}$] or [*Zp3-cre*, *Tet3*$^{F/–}$] female mice. FG oocytes at the GV stage were stained with anti-mTET3(C) (red in merged images), and nuclei were revealed by staining of DNA with Sytox (green in merged images). Dashed circles indicate the rim of the oocyte.

Supplementary Figure S2. Incorporation of 5EU in zygotes is inhibited by treatment with α-amanitin.

Zygotes derived from oocytes of [*Zp3-cre*, *Tet3*$^{F/+}$] female mice were pulse-labeled with 5EU from 4 to 12 hpi in the absence (Ctrl) or presence of α-amanitin (60 μg/ml). Incorporated 5EU was then observed (red in merged images). Pronuclei were revealed by staining of DNA with Sytox (green in merged images). Dashed circles indicate the rim of the zygote.

Supplementary Figure S3. Cell cycle progression of zygotes is not obviously influenced by loss of maternal TET3.

(a) Representative images showing accumulation of incorporated 5EdU (red in merged images) in zygotes derived from oocytes of [*Zp3-cre*, *Tet3*$^{F/+}$] or [*Zp3-cre*, *Tet3*$^{F/–}$] female mice and pulse-labeled with 5EdU for the indicated time periods. Pronuclei were revealed by staining of DNA with Sytox (green in merged images). (i) and (ii) are representative images of zygotes in which the signal for 5EdU was detected (27%) or not detected (73%), respectively. Dashed circles, ♂, and ♀ indicate the rim of the zygote, the paternal pronucleus, and the maternal pronucleus, respectively. *Each pair of images was obtained from two different planes of the same zygote. (b) Zygotes derived from oocytes of [*Zp3-cre*, *Tet3*$^{F/–}$] (4–5 hpi, n = 10; 5–6 hpi, n = 13; 9–10 hpi, n = 11) or [*Zp3-cre*, *Tet3*$^{F/+}$] (4–5 hpi, n = 9; 5–6 hpi, n = 9; 9–10 hpi, n = 15) female mice and pulse-labeled with 5EdU were examined for determination of the percentage in S phase. The P values were determined with the one-sided Fisher's exact probability test. (c) Zygotes derived from oocytes of [*Zp3-cre*, *Tet3*$^{F/+}$] female mice were pulse-labeled with 5EdU from 4 to 10 hpi in the absence (Ctrl) or presence of

aphidicolin (5 μg/ml). Incorporated 5EdU was then observed (red in merged images), and pronuclei were revealed by staining of DNA with Sytox (green in merged images). Dashed circles indicate the rim of the zygote.

Supplementary Figure S1

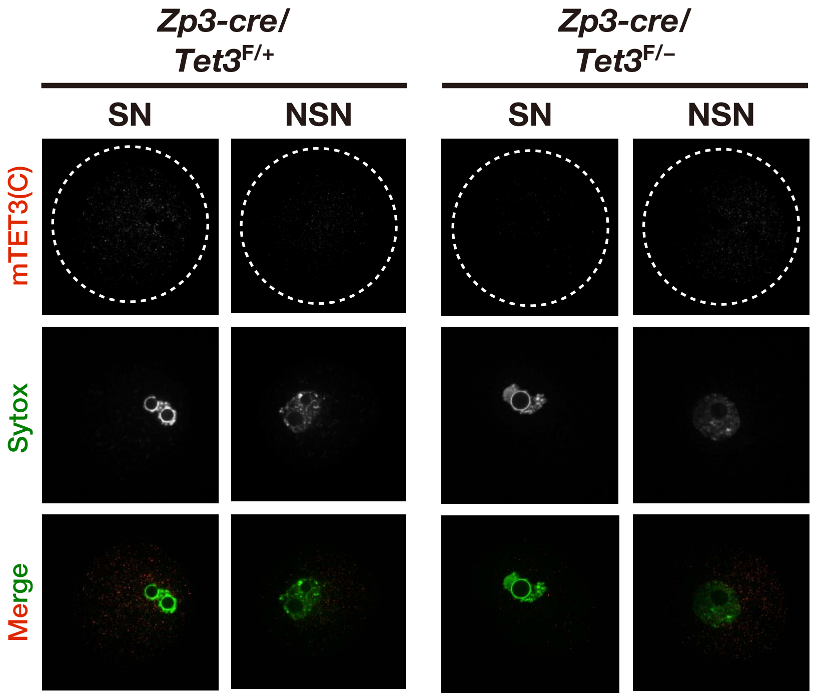

Supplementary Figure S2

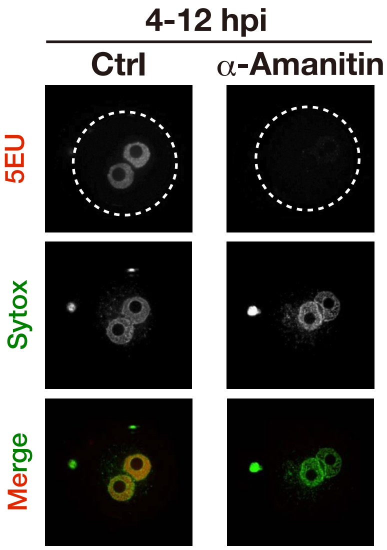

Supplementary Figure S3

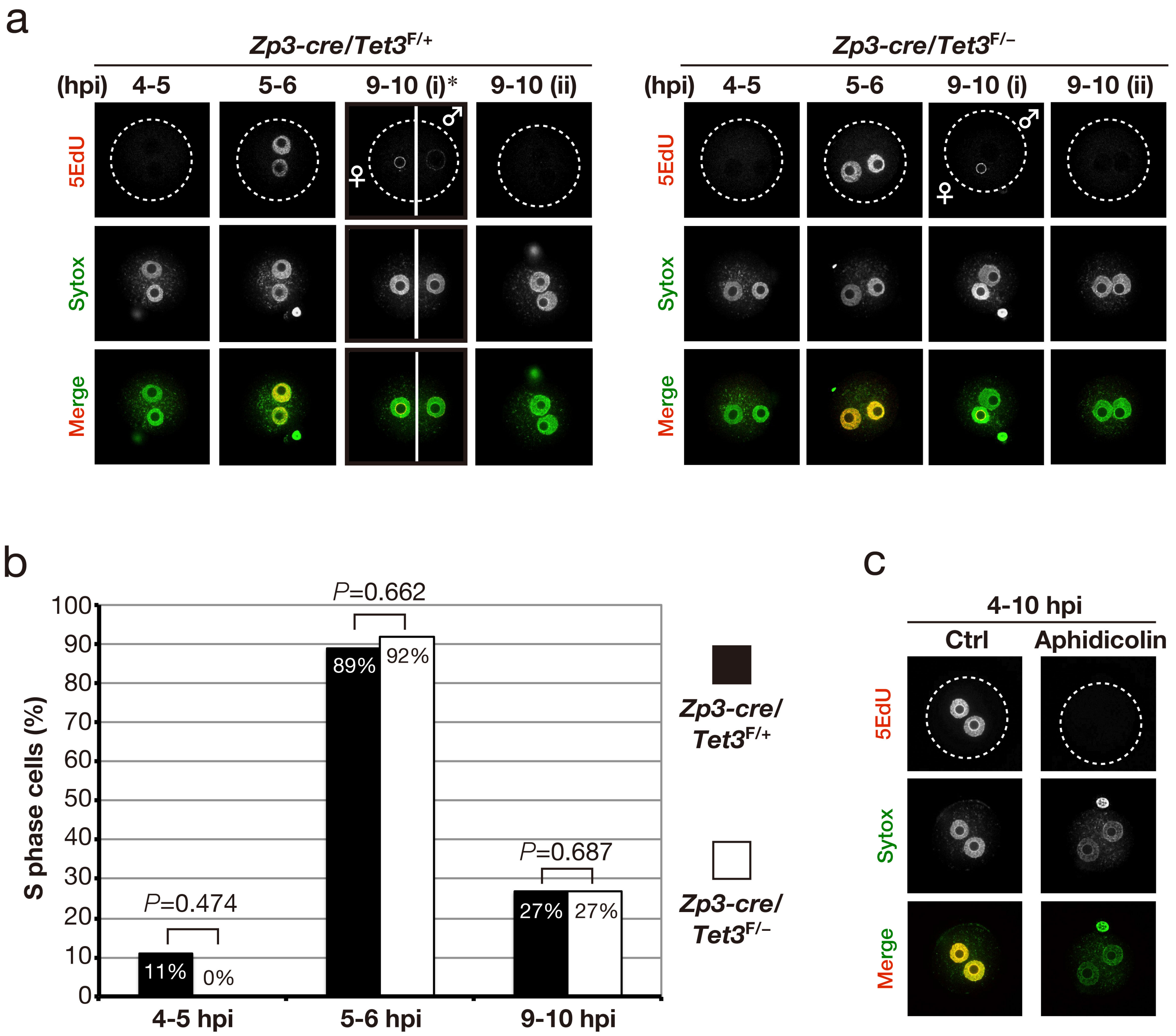

Supplement: Supplementary Information [file srep15876-s1.pdf]
